# Prospects of Intravenous Coenzyme Q10 Administration in Emergency Ischemic Conditions

**DOI:** 10.3390/life14010134

**Published:** 2024-01-17

**Authors:** Elena I. Kalenikova, Evgeniya A. Gorodetskaya, Oxana V. Povarova, Oleg S. Medvedev

**Affiliations:** 1 Faculty of Medicine, Lomonosov Moscow State University, 119991 Moscow, Russia; eikaleni@fbm.msu.ru (E.I.K.); gorodets@fbm.msu.ru (E.A.G.); medvedev@fbm.msu.ru (O.S.M.); 2 National Medical Research Center of Cardiology of the Ministry of Health of the Russian Federation, Laboratory of Experimental Pharmacology, 121552 Moscow, Russia

**Keywords:** coenzyme Q10, ubiquinol, ubiquinone, brain ischemia, myocardial infarct, intravenous administration

## Abstract

Coenzyme CoQ10 (CoQ10) is an endogenous lipid-soluble antioxidant that effectively protects lipids, proteins, and DNA from oxidation due to its ability to undergo redox transitions between oxidized and reduced forms. Various oxidative stress-associated infectious and somatic diseases have been observed to disrupt the balance of CoQ10 concentration in tissues. As a high molecular weight polar lipophilic compound, CoQ10 exhibits very limited oral bioavailability, which restrains its therapeutic potential. Nevertheless, numerous studies have confirmed the clinical efficacy of CoQ10 therapy through oral administration of high doses over extended time periods. Experimental studies have demonstrated that in emergency situations, intravenous administration of both oxidized and reduced-form CoQ10 leads to a rapid increase in its concentration in organ tissues, offering protection for organ tissues in ischemic conditions. This suggests that the cardio- and neuroprotective efficacy of intravenously administered CoQ10 forms could present new opportunities in treating acute ischemic conditions. Based on these findings, the review provides reasoning supporting further research and implementation of CoQ10 dosage forms for intravenous administration in emergency situations into clinical practice.

## 1. Introduction

Coenzyme Q10 (CoQ10) is a multifunctional high molecular weight lipophilic compound that naturally exists in all cells of living organisms.

It was first isolated from beef heart tissue in 1957 by a group of scientists led by Crane at Madison University and was named ubiquinone [1]. The CoQ10 molecule consists of a benzoquinone ring with a side chain composed of 10 hydrocarbon (isoprene) units. Due to its hydrophobic and polar nature, this compound has low bioavailability when administered orally.

## 2. Biological Functions of Coenzyme Q10

CoQ10 is known to be ubiquitously present in all human tissues, predominantly in high metabolic activity organs like the heart, kidneys, liver, and muscles, where it exists primarily as ubiquinol [2]. One of its most important functions is to participate in the respiratory chain of mitochondria due to its ability to receive and give electrons and protons. Participating in ATP synthesis, CoQ10 regulates the cellular functions in the body and primarily in organs with a high energy demand. Moreover, CoQ10 affects mitochondrial functions, modulating transmembrane mitochondrial pores, and normalizing mitochondrial fission through its control of dynamin related protein 1 (Drp1) and mitochondrial adaptor fission 1 (Fis1) [3]. It also regulates the activity of nuclear factor-κB (NF-kB), impacting the activity of inflammasomes and reducing the production of pro-inflammatory cytokines. CoQ10 is involved in apoptosis and mitophagy processes, in particular in regulation of the activity of apoptosis proteins [4].

Additionally, CoQ10 regulates the activity of nitric oxide synthase (NOS) [4]. Studies have demonstrated that coenzyme Q10 exhibits both inhibitory effects on inducible nitric oxide synthase (iNOS) and endothelial nitric oxide synthase (eNOS) [5], and stimulatory effects, as confirmed in an experimental rat model of seizures with the activation of constitutive NOS [6]. Notably, in a model of acrylonitrile-associated endothelial dysfunction in rat aortas, a stimulatory effect of CoQ10 on eNOS was also observed [7].

CoQ10 has been reported to affect endothelial function in patients with type 2 diabetes mellitus and coronary heart disease, resulting in accelerated nitroglycerin-mediated dilatation and increased extracellular superoxide dismutase activity [8]. CoQ10 has also been found to extend the duration of NO effects by reducing oxidative stress marker levels, accompanied by increased superoxide dismutase activity and decreased peroxynitrite formation rates [9]. In vitro studies have further established CoQ10’s role in stimulating angiogenesis among precursors and inhibiting glucose-mediated apoptosis of endothelial cells [10,11].

Research conducted on isolated rat vessels has also revealed that the vasodilating effect of coenzyme Q10 on the rat aorta depends on nitric oxide (NO). Upon intravenous administration of CoQ10 to rats, the NO-dependent relaxation of aortic rings induced with acetylcholine notably improved comparable to the effect of L-arginine, an eNOS substrate. These findings have shown for the first time that exogenous coenzyme Q10, when administered intravenously, can rapidly enhance NO-dependent vascular vasodilation, likely owing to CoQ10 accumulation in the vascular endothelium [12]. The endothelial function enhancement may explain, at least partially, CoQ10’s beneficial effects in cardiovascular diseases associated with endothelial dysfunction and its anti-ischemic properties.

Moreover, data supporting the influence of CoQ10 on endothelial dysfunction, one of the triggers in atherosclerosis, are presented [13].

**Figure 1:**
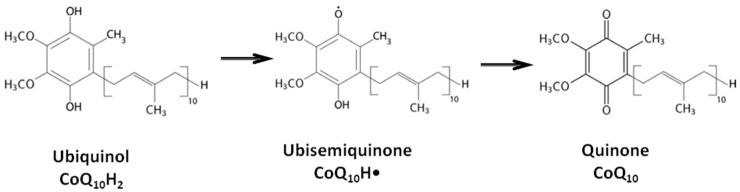
Oxidation of ubiquinol to ubiquinone.

Functioning as an endogenous lipid-soluble antioxidant, CoQ10 effectively protects lipids, proteins, and DNA from oxidation by undergoing redox transitions between its oxidized and reduced forms (see Figure 1).

The antioxidant effect of CoQ10 occurs primarily due to the action of its reduced form (ubiquinol) safeguarding cell membrane phospholipids and intracellular organelles from oxidation. Both the oxidized form (ubiquinone) and the semi-reduced form (ubisemiquinone) of CoQ10, along with the reduced forms of α-tocopherol and vitamin C, contribute to maintaining the cell’s redox equilibrium and regulating the physico-chemical properties of the membrane [2].

Additionally, when orally administered, CoQ10 potentially modulates the intestinal microbiota, leading to increased antioxidant production, specifically molecular hydrogen. CoQ10 can also affect the production of butyric acid, potentially enhancing the protective barrier function of the intestine [14].

## 3. Efficacy of Coenzyme Q10 Therapy When Administered Orally

Coenzyme Q10 deficiency, whether primary (congenital) or secondary (acquired), manifests inclinical symptoms affecting various organs and systems, including neurological disorders, cardiovascular and muscular pathologies, and nephropathy [15]. Primary CoQ10 deficiency replacement therapy necessitates high doses of CoQ10, with a minimum of 2.4 g/day in adults and not less than 30 mg/kg/day in children over an extended period. The clinical efficacy of CoQ10 has been confirmed in many of clinical studies conducted to date, including recent publications [8,13,15,16,17,18].

However, a major challenge with CoQ10 replacement therapy is its low oral bioavailability, estimated at no more than 2%, according to experimental data obtained in rats [19]. Although solubilized forms exhibit 2–3 times greater bioavailability, it remains insufficient [20,21,22]. Initially, the focus was on developing of CoQ10 formulations with enhanced bioavailability using non-polar agents as delivery systems due to their advantages such as increased drug solubility in the intestine, activation of drug lymphatic transport or altered drug transport, and distribution in enterocytes.

Current interest lies in developing water-soluble CoQ10 forms to address common disadvantages of lipid-based formulations concerning dispersion speed, emulsification rate, particle size, and drug precipitation upon dispersion. Initially, lipid-free CoQ10 nanoforms, CoQ10 micellar solutions, and hydrophilic complexes incorporating CoQ10 were developed [23]. Pharmaceutical products with enhanced CoQ10 solubility, manufactured by Tishcon (Nanodispersion and Solubilisate) and by Pharma Nord ApS (Solubilisate, an oil-based formulation) are available on the market [18].

Studies investigating the pharmacokinetics of different CoQ10 dosage forms (water-soluble syrup of CoQ10 and ubiquinone/ubiquinol capsules) in elderly patients, representing those with developing secondary CoQ10 deficiency, confirmed the higher bioavailability of CoQ10 in the syrup form [24]. Simultaneously, the redox status of CoQ10 in patients’ plasma remained independent of the drug administered, whether ubiquinone or ubiquinol, corroborating previous animal studies [25].

The majority of published experimental animal studies and all clinical studies evaluating the efficacy and safety of CoQ10 products typically follow protocols with oral administration of various ubiquinone or ubiquinol dosage forms.

Numerous studies confirm that CoQ10 supplementation over a wide range of doses is safe and well tolerated. Notably, there have been no reported serious side effects leading to therapy discontinuation. Most observed side effects are primarily associated with the route of CoQ10 administration, and such effects manifest predominantly in the gastrointestinal tract [16,22,26,27]. Just a few instances of adverse reactions, such as platelet dysfunction, allergic reactions, lymphadenopathy, and cheilitis, have been reported [16,27]. Comparative analyses have shown no significant differences in the rate of side effects—such as lumbarpuncture-induced headache, back pain, and lumbarpuncture-induced nausea—between placebo groups and those receiving different dosages of ubiquinol at 900 mg, 1200 mg, and 1500 mg [27].

Consistently, all studies affirm the high safety profile of CoQ10. Several meta-analyses have focused on CoQ10’s efficacy in cardiovascular diseases, such as chronic heart failure (CHF) and arterial hypertension.

The multicenter, randomized, double-blind Q-Simbio trial was carried out in patients with moderate to severe CHF (231 participants) and who received treatment in European centers. The analysis revealed no significant differences in the primary endpoints (3 months of follow-up) compared to the placebo group. However, secondary endpoint analysis (2 years) revealed substantial improvements in all-cause mortality and cardiovascular mortality, a decrease in a number of hospitalizations, and an enhanced left ventricular ejection fraction, particularly with better therapy adherence [26]. In a Cochrane meta-analysis encompassing 11 randomized clinical trials of CoQ10 in patients with heart failure (1573 participants), it was concluded that CoQ10 administration reduces overall mortality and hospitalization duration due to HF, with minimal effects on left ventricular (LV) ejection fraction, heart attack risk, risk of stroke, and exercise tolerance [28].

A meta-analysis of eight clinical trials with a total of 327 patients undergoing cardiac surgery with cardiopulmonary bypass, revealed the efficacy of preventive CoQ10 administration with a duration from 6 to 14 days. CoQ10 administration significantly reduces the proportion of patients who require inotropic drugs after surgery and significantly reduces the rate of ventricular arrhythmias after surgery [29]. It is worth noting that a single preventive oral dose of CoQ10 did not yield any therapeutic effect [30].

A meta-analysis incorporating 50 randomized clinical trials of CoQ10 in cardiovascular patients (2794 participants), revealed significant changes in the lipid profile: a decrease in total cholesterol, low-density lipoprotein cholesterol (LDL-C), triglyceride (TG) and an increase in high-density lipoprotein cholesterol (HDL-C) [31]. The most pronounced effects were observed at the dose of 400–500 mg/day. Similarly, a meta-analysis of 8 randomized clinical trials conducted on patients with coronary heart disease (267 participants) demonstrated a significant decrease in total cholesterol and an increase in HDL-C levels without affecting TG, LDL-C, or lipoprotein A [32].

A meta-analysis encompassing 31 randomized clinical trials with 1517 subjects, evaluated the anti-inflammatory effects of CoQ10 regarding C-reactive protein (CRP), tumor necrosis factor-alpha (TNF-α), and interleukin 6 (IL-6). It revealed a significant anti-inflammatory effect of CoQ10 at a daily dose of 300–400 mg [33].

Regarding statin-induced myopathy, results are somewhat conflicting. In a meta-analysis of 12 randomized clinical trials, a positive effect of CoQ10 administration was identified [34]. At the same time, a meta-analysis of 5 randomized clinical trials with 321 patients did not confirm substantial improvements to patients’ conditions [35].

Evaluation of CoQ10’s hypotensive efficacy in patients with arterial hypertension [36] and metabolic diseases [37] via meta-analyses demonstrated its ability to reduce systolic [36,37] and diastolic blood pressure [37].

Regarding the use of CoQ10 in various neurological diseases to assess its impact on neurologic symptoms, multiple reviews have been presented [2,4,18]. The analysis of CoQ10 efficacy in clinical neurology practice, in comparison to experimental results, highlights the necessity for higher doses and longer courses of CoQ10 therapy [4]. In patients with acute cerebral blood flow disorders, oral administration of CoQ10 at a daily dose of 300 mg significantly improved Mini-Mental State Examination (MMSE) and National Institutes of Health Stroke Scale (NIHSS) scores [38]. Furthermore, a meta-analysis examining CoQ10 use in migraine patients observed a reduction in both the duration and frequency of migraine attacks [39].

Additionally, CoQ10 has been shown to be effective against diseases and pathological conditions such as diabetic angiopathy and nephropathy, insulin resistance, multiple systemic atrophy, immunodeficiency states, Bart’s syndrome, familial hypercholesterolemia, and fibromyalgia [23].

Ongoing clinical trials continue to explore the efficacy of CoQ10 in various pathological conditions (Table 1) [40].

The limited clinical efficacy of CoQ10 observed in several studies may be attributed to its low oral bioavailability and short duration of therapy, which result in low CoQ10 levels in both plasma and tissues and thus hinder CoQ10 from achieving its therapeutic potential. For instance, in a study involving patients experiencing clinical cardiac arrest, a seven-day course of CoQ10, despite a dosage of 300 mg taken twice daily resulting in increased plasma CoQ10 levels, failed to demonstrate an improvement in neurological and biochemical parameters [58]. Similarly, a meta-analysis of clinical studies investigating CoQ10 influence on blood pressure (50 participants) revealed that a three-week administration period was insufficient to have a significant impact on blood pressure and heart rate, leading the researchers to advocate for longer-duration studies [59].

## 4. Results of Intravenous Coenzyme Q10 Administration in Experimental Models In Vivo

In emergency situations like myocardial infarction or ischemic stroke, where acute CoQ10 deficiency is evident, alternative routes of administration, particularly intravenous (IV) delivery, may be more effective [60].

The major advantage of intravenous administration is CoQ10’s rapid penetration into tissues, which significantly increases CoQ10 tissue concentration and enables the achievement of substantial anti-ischemic potential during acute ischemia and reperfusion.

An acute oxygen deficiency is at the core of ischemic damage in any organ, which leads to disruption of the normal function of mitochondria, the main consumers of oxygen and ATP generators. CoQ10, being an integral component of the mitochondrial respiratory chain, facilitates electron transfer between complexes I and III, as well as II and III, and ensures normal mitochondrial function [61,62].

An increased CoQ10 tissue level in the form of ubiquinol, administered via intra-venous injection, can effectively restrict the escalating electron leakage from the mitochondrial respiratory chain during ischemia and reduce the formation of harmful ROS, which damage the cell’s structural components. Notably, by administering coenzyme Q10 during ischemia, we safeguard mitochondrial function in the “risk zone” and prevent the progression of irreversible reactions leading to extensive cellular and tissue death.

Vasospasm, being a consequence of oxidative stress, exacerbates tissue damage [63]. In brain tissue, vasospasm accompanies “cortical spreading depolarization” (SD) [64]. Rapid vasodilation resulting from CoQ10’s intravenous administration impacts endothelial function, potentially reducing post-ischemic vasospasm and subsequent lesion volumes [12].

During ischemia coupled with tissue reperfusion, in addition to the aforementioned mechanisms of anti-ischemic protection, CoQ10’s antioxidant properties start playing a pivotal role [65].

Another pathogenetic mechanism of ischemic injury is inflammation associated with oxidative stress. CoQ10’s anti-inflammatory effects manifest at the onset of ischemia. These effects are attributed to CoQ10’s membrane-stabilizing and antioxidant actions, as well as CoQ10’ inhibition of inflammation-related cytokine gene expression and the elevation of anti-inflammatory biomarkers levels [33,66].

In addition to the previously mentioned mechanisms, CoQ10’s anti-ischemic action involves anti-apoptotic effects, increasing the levels of ubiquitin proteins and enhancing autophagy. Furthermore, CoQ10 decreases the levels of angiotensin-converting enzyme (ACE), preventing myocardial remodeling [67].

A meta-analysis of 6 published preclinical studies involving 116 animals with myocardial infarction modeling reperfusion confirmed the cardioprotective efficacy of CoQ10. On average, regardless of the model of myocardial ischemia or animal species used, CoQ10 administration resulted in a reduction of the infarct area by 11.36% compared to control groups [65].

However, research on the efficacy of CoQ10 through intravenous administration remains limited [68], primarily due to the lack of dosage forms approved for clinical use [69,70,71]. Animal studies have explored micellar or liposomal forms of CoQ10 [71] and solubilizers, such as HCO-60 (polyoxyethylene hydrogenated castor oil-60) [72] or caspofungin [69]. Intracoronary administration of liposomal CoQ10 to rabbits at a dosage of 36 mg before a 30-min occlusion of the left coronary artery followed by 3 h of reperfusion resulted in a more than twofold reduction in the myocardial necrosis zone [73]. In rats, intramuscular administration of coenzyme Q10 at a dose of 20 mg/kg for 7 days prior to coronary artery occlusion led to a 57% reduction in the infarct zone area. This treatment normalized hemodynamic parameters and also lowered the levels of inflammatory and oxidative stress markers [74].

A novel CoQ10 dosage form designed for intravenous administration, based on solubilized ubiquinol, has successfully completed preclinical studies [75]. 

The pharmacokinetics of this new form of ubiquinol was studied in rats in comparison with ubiquinone, both forms having a similar excipient composition [76]. Both kinetic curves were found to be biphasic, with a comparable initial decrease rate in plasma concentration during the first hours after injection. However, ubiquinone demonstrated significantly higher plasma CoQ10 levels between 24 and 96 h after administration and equalized with ubiquinol by the eighth day after administration. The area under the curve (AUC192 h) for ubiquinone was 1.5 times higher than that for ubiquinol. Accordingly, the total clearance of ubiquinol was 1.5 times higher than that of ubiquinone. 

A single intravenous injection of CoQ10 at a dosage of 30 mg/kg leads to an immediate increase in its concentration in blood plasma by several times. This elevated concentration persists for several hours, significantly exceeding the CoQ10 concentration in organ tissues. The transfer of CoQ10 from plasma to organs is accelerated, and a significant increase in CoQ10 concentration in tissues is observed as early as 15 min after administration. The tissue distribution of CoQ10 after injection showed similar patterns for both forms: in 15 min, its concentration increased by 2.5 times in the heart, by 1.7–2.0 times in the brain, and by 3.5 times in the kidneys. CoQ10 concentration remained elevated (by 70–50%) for at least 48 h upon injection. In the liver, CoQ10 accumulated gradually, peaking in 1–2 days, with maximum levels not significantly different between the forms, exceeding baseline levels by 17–23 times. Even on the eighth day, CoQ10 concentration in the liver remained substantially (7–10 times) higher than baseline levels.

Comparative analysis of AUC and total body clearance (Clt) values between ubiquinone and ubiquinol revealed that ubiquinol was eliminated faster from blood plasma, and reached maximum concentration in the liver earlier. Notably, the total CoQ10 amount accumulating in the liver remained consistent regardless of administration in oxidized or reduced form. The accumulation and subsequent secretion of CoQ10 into the blood via lipoproteins might contribute to its sustained elevated concentration levels in plasma and tissues over time [76].

Following intravenous ubiquinol injection, CoQ10 redox status—defined as the proportion of the reduced form in the total pool—in blood plasma remained constant during the initial 48 h at the level of 92%. After the injection of the CoQ10 oxidized form in blood, a gradual reduction occurred, with the ubiquinol proportion reaching approximately 89% by the end of the first day. This level is assumed to reflect the endogenous CoQ10 redox balance in rat blood plasma, analogous to human levels [25].

Notably, in the myocardium and brain, the proportion of the CoQ10 reduced form was significantly lower than in plasma, remaining constant throughout the entire observation period: prior to ubiquinol administration, during the initial 96 h of elevated tissue concentration, and after returning to baseline levels by the eighth day. Thus, CoQ10 redox status is specific for each tissue of the organism and remains unchanged when CoQ10 tissue levels are increased as a result of intravenous administration. The revealed constancy of CoQ10 redox status, irrespective of the variations in absolute concentrations, suggests the presence of mechanisms governing CoQ10 redox status. Clear differences in CoQ10 redox status in blood plasma and organs indicate partial oxidation of ubiquinol to reach the level of endogenous redox balance upon the transfer from blood into organ tissues, involving the drug in local redox processes [25].

The rapid replenishment of CoQ10 tissue concentration and enhanced antioxidant capacity through intravenous administration, as revealed in pharmacokinetic studies, holds promise for acute ubiquinone deficiency, particularly in urgent ischemic conditions. The cardio- and neuroprotective efficacy of IV CoQ10 has been affirmed in experimental models of myocardial and brain infarction in animals. Pharmacokinetic research of intravenous administration at a dose of 30 mg/kg supports the use of such doses in experimental pathology models [77,78,79,80,81,82,83].

The cardioprotective effects of preventive ubiquinone administration has been demonstrated in a rat model of ischemia–reperfusion. Animals were intravenously injected with ubiquinone or saline 30 min before coronary artery occlusion. After 30 min of ischemia and 120 min of reperfusion, the area of left ventricular infarction and the level of CoQ10 in the myocardium were assessed. At reperfusion initiation, arrhythmias were observed in 8 out of 9 rats receiving saline, contrasting with only 2 out of 9 rats receiving ubiquinone. In the ubiquinone group, arrhythmias appeared later and were of shorter duration compared with untreated animals. Additionally, in the group of animals receiving ubiquinone, the CoQ10 concentration was twice as high in the left ventricle, and the infarct area was one-third less than in the untreated group. Correlation analysis revealed that higher CoQ10 concentrations in myocardial tissue corresponded to smaller infarct sizes [77].

Further investigations on the cardioprotective effects of ubiquinone administration in irreversible myocardial ischemia models revealed promising results. In rats, ubiquinone was administered intravenously 10 min after coronary artery occlusion. By day 21 after myocardial infarction, CoQ10 concentration in plasma, left ventricle, and liver in these animals was higher than in untreated rats by 87%, 23%, and 1042%, respectively. The size of the myocardial necrosis zone was smaller, and postinfarction hypertrophy was less severe in rats treated with CoQ10. These rats had higher values of stroke volume (by 24.6%), stroke work (by 34.9%), cardiac output (by 37.8%), ejection fraction (by 35.7%), contractility (by 22.5%), and lower end-diastolic pressure (by 25.8%) than untreated animals [78]. CoQ10 was shown to have cardioprotective efficacy when administered within 60 min after occlusion [79].

The cardioprotective efficacy of intravenous ubiquinol administration was demonstrated in the same model of irreversible myocardial ischemia. Intravenous administration of ubiquinol (10 mg/kg) within 10 min after coronary artery occlusion resulted in a significant reduction of left ventricular myocardial aneurysm size on the 21st day (13.19% vs. 31.55% for treated and untreated groups, respectively). It also prevented the development of left ventricular myocardial hypertrophy and helped to control the decrease of cardiac pumping function. Additionally, in the treated animal group, an inverse correlation between CoQ10 concentration in the myocardium and interventricular septal thickness (r = −0.672, *p* < 0.05) was found, which emphasizes its role in controlling post-infarction damage. This result was comparable to the efficacy observed with intravenous administration of a higher dose (30 mg/kg) of oxidized CoQ10 [80].

Thus, a single intravenous injection of both oxidized and reduced forms of CoQ10 before or during myocardial ischemia elevates its concentration in the myocardium and exerts cardioprotective effects, minimizing the infarct zone, controlling myocardial hypertrophy, and enhancing functional heart characteristics.

**Figure 2:**
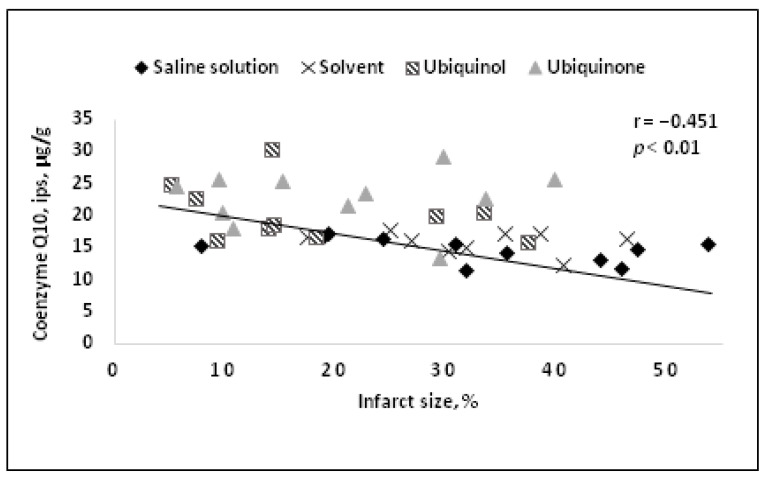
Correlation between area of the infarct region and CoQ10 tissue concentration in the ipsilateral hemisphere [83].

Moreover, the neuroprotective CoQ10 effect has been demonstrated viaintravenous administration in models of reversible and irreversible brain ischemia [81,82,83], and the correlations between CoQ10 tissue concentration and damage zone sizes have been established (Figure 2) [83].

In male Wistar rats, reversible ischemia was induced via middle cerebral artery occlusion for 60 min followed by reperfusion. A single intravenous injection of ubiquinone or saline was administered 15 min before reperfusion. Sensory and motor functions, cerebral infarct volume, and CoQ10 concentration were assessed either 1 or 7 days later. Cerebral ischemia resulted in a significant decrease in endogenous CoQ10 concentration in both hemispheres. Intravenous ubiquinone injection increased its concentration in both hemispheres up to the concentration level in a sham group, and significantly improved neurological status and reduced the volume of cerebral infarction by 67% at day 1 and by 35% at day 7 following artery occlusion [81].

Similarly, in experimental models of irreversible cerebral ischemia resulting from middle cerebral artery occlusion, the neuroprotective efficacy of ubiquinone has also been demonstrated. Ischemic stroke was accompanied by a decrease in CoQ10 levels in both ipsilateral and contralateral hemispheres. Intravenous ubiquinone administration increased its concentration in both hemispheres. At 24 h, the neurological status of animals that received ubiquinone injection within 60 min after the onset of ischemia, as compared to untreated animals, was significantly better, mainly due to motor function improvement; also, the volume of brain necrosis was half as great [82]. Thus, it was shown that intravenous administration of ubiquinone in models of transient and chronic cerebral ischemia is accompanied by CoQ10 permeation into the brain and the achievement of neuroprotective effect.

**Figure 3:**
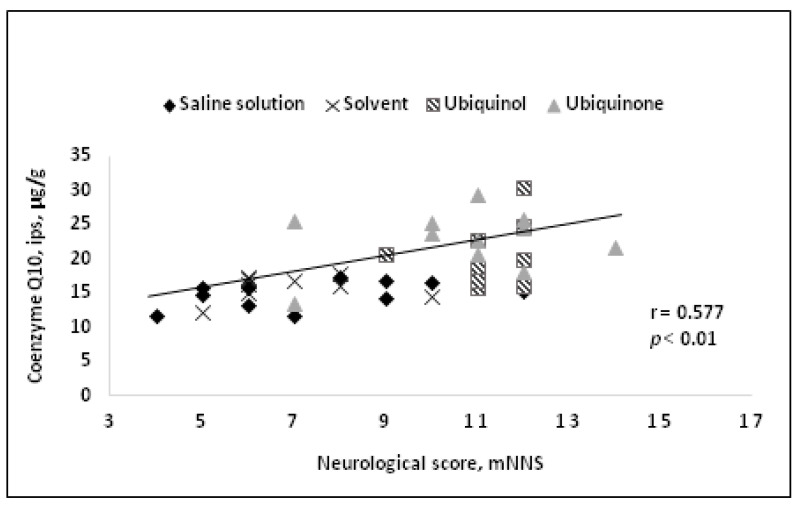
Correlation between neurological deficit and CoQ10 tissue concentration in the ipsilateral hemisphere [83].

A comparative study of neuroprotective efficacy between ubiquinone and ubiquinol in a reversible cerebral ischemia model during one day administering the drugs intravenously 15 min before reperfusion was carried out [83]. On the first day after the onset of ischemia, a decrease in mortality up to 10% compared to 57% in the control group, an improvement to neurological status, and brain necrosis abatement were revealed. At the same time, CoQ10 concentration in the brain tissue was found to correlate with both the size of the necrotic region and the neurological status of the animals (Figure 2 and Figure 3).

A significant decrease in CoQ10 tissue concentration after ischemia in untreated animals in both ipsilateral and contralateral hemispheres was noted as early as by the end of the first day, followed by further CoQ10 concentration decrease by the end of the fourth day. Intravenous administration of both ubiquinol and ubiquinone in treated animals helped to increase CoQ10 concentration levels within 24 h compared with a sham group. By the day 4 after ubiquinol injection, the CoQ10 concentration in both hemispheres in animals remained at the level observed in the sham group.

In the same study, the neuroprotective efficacy of CoQ10 at 4 days was evaluated using ubiquinol as an example. The mortality of animals in the control group on day 4 reached 80%, compared to 20% mortality rate in the group treated with ubiquinol. The neurological deficit in ubiquinol-administered animals did not worsen, unlike in the untreated group. MRI assessment was used to evaluate changes in the size of the lesion in each animal during 4 days. Within this period, there was an almost twofold increase in the area of the brain lesion in the control group. In the group of animals treated with ubiquinol injection, the size of necrotic region did not increase compared to the size of necrotic region by the end of the first day.

Using the same model of focal rat brain ischemia and an intravenous route of CoQ10 administration at the same dose of 30 mg/kg, the following was demonstrated: improved neurological status of treated animals in terms of sensory–motor functions, decreased necrosis, enhanced viability of blood–brain barrier, and reduced brain edema. All of the above findings are consistent with our results. The neuroprotective efficacy of CoQ10 is attributed to the reduction of proinflammatory cytokines and an involvement in molecular mechanisms related to miR-149-5p gene expression [66].

Thus, a single intravenous injection of either oxidized or reduced forms of CoQ10 in experimental cerebral ischemia increases the CoQ10 concentration in both cerebral hemispheres. This manifests considerable neuroprotective effects, including mitigating the necrotic region, preventing its expansion, and preserving neurological status. The observed inverse correlation between the lesion size and CoQ10 concentration in tissues further supports the neuroprotective potential of CoQ10.

These experimental findings underscore the potential of intravenous CoQ10 administration to offer substantial cardio- and neuroprotective effects in cases of heart and brain ischemia, irrespective of CoQ10 redox state.

## 5. Conclusions

The administration of both oxidized and reduced forms of CoQ10 intravenously in the experimental models demonstrated significant protection for heart and brain tissues affected by ischemic events. These results emphasize the necessity to develop intravenous CoQ10 dosage forms and warrant further clinical studies to assess their efficacy.

## Figures and Tables

**Table 1:** Clinical trials of coenzyme Q10 (planned and published) from 2021 to 2023 registered in the Cochrane Library database.

Disease	Name of Study	Daily Dose of CoQ10, Duration of Administration	Results	Country, Year of Registration
Ischemic stroke	Evaluation of the effects of coenzyme Q10 on stroke	600 mg/day for 30 days, per os	Not yet published	Iran, 2021 [41]
Children with tetralogy of Fallot	The safety and efficacy of preoperative oral supplementation of coenzyme Q10 in improving postoperative cardiac function in children with tetralogy of Fallot (pulmonary atresia): a preliminary study	Different dosages before surgery: 2.5 mg; 5 mg; 10 mg; 20 mg. Duration not specified, per os	Not yet published	China, 2019 [42]
Statin-induced myalgia	Coenzyme Q10 and tolerability of simvastatin in subjects with a history of statin-induced myalgia	200 mg/day for about 3 months, per os	Not yet published	New Zealand, since 2005 (update 2020) [43]
Acute coronary syndrome (ACS)	Influence of ubiquinol on angina severity and dyspnea in patients with acute coronary syndrome	200 mg/day of ubiquinol for 8 weeks	Ubiquinol addition to optimal medical therapy (OMT) after ACS has a highly significant effect on improving clinical outcomes and patients’ quality of life through greater reductions in angina frequency, physical limitations, and dyspnea severity. This suggests an effective and safe strategy for optimizing therapeutic outcomes and secondary prevention	Iraq, 2023 [44]
Metabolic syndrome	Effects of curcumin and/or coenzyme Q10 supplementation on metabolic control in subjects with metabolic syndrome: a randomized clinical trial	60 mg/day for 12 weeks, per os	CoQ10 showed no therapeutic effects	Iraq, 2021 [45]
Acute myocardial infarction	A randomized controlled trial on the effect of Co Q10 on vascular endothelial and cardiac function after percutaneous coronary intervention therapy for acute myocardial infarction	Dose and duration of administration not specified, per os	Not yet published	China, 2021 [46]
Heart failure with preserved ejection fraction (HFpEF)	Coenzyme Q10 in the treatment of heart failure with preserved ejection fraction: a prospective, randomized, double-blind, placebo-controlled trial	300 mg/day of ubiquinol for 4 months, per os	In this pilot trial in elderly patients with HFpEF, treatment with CoQ10 did not significantly affect echocardiographic indices of diastolic function and serum N-terminal pro-B-type natriuretic peptide (NT-proBNP) levels.	Israel, 2021 [47]
Prevention of high-altitude heart disease	Effect of Coenzyme Q10 on prevention of high-altitude heart disease and improvement of cardiac function	Different dosages, dose and duration not specified, per os	Not yet published	China, 2021 [48]
Metabolic associated fatty liver disease (MAFLD)	Effect of CoQ10 on the outcome of MAFLD patients	200 mg/day for 12 weeks, per os	Not yet published	Egypt, 2023 [49]
Nonalcoholic steatohepatitis	Comparative clinical study to evaluate the efficacy and safety of rosuvastatin vs. CoQ10 on nonalcoholic steatohepatitis	100 mg/day for 3 months, per os	Not yet published	Egypt, 2023 [50]
Dyslipidemia	Coenzyme Q10 supplementation improves adipokine profile in dyslipidemic individuals: a randomized controlled trial	120 mg/day for 24 weeks, per os	This study shows that CoQ10 ameliorates glucolipid profile and adipokines dysfunction in dyslipidemic patients in 24 weeks’ intervention. The beneficial effect of CoQ10 on glucolipid profile was mediated by adiponectin.	China, 2022 [51]
Diabetic nephropathy	Effects of coenzyme Q10 supplementation on renal function parameters in patients with diabetic nephropathy: a randomized controlled trial	100 mg/day for 6 months, per os	This study found that daily administration of 100 mg CoQ10 improved the mean proteinuria, glomerular filtration rate (GFR) and creatinine levels in patients with diabetic nephropathy.	Iran, 2022 [52]
Acute herpes zoster	To evaluate the analgesic effect of Co Q10 in acute herpes zoster	100 mg/day for 4 weeks, per os	Not yet published	India, 2023 [53]
Juvenile idiopathic arthritis	Coenzyme Q10 in juvenile idiopathic arthritis patients	100 mg/day for 3 months, per os	Not yet published	Egypt, 2023 [54]
Polycystic ovary syndrome (PCOS)	The effects of coenzyme Q10 supplementation on metabolic profiles and parameters of mental health in women with polycystic ovary syndrome	100 mg/day for 12 weeks, per os	12-week supplementation of CoQ10 to women with PCOS showed beneficial impact on the scores of Beck Depression Inventory(BDI), Beck Anxiety Inventory(BAI), high-sensitivity C-reactive protein(hs-CRP), total testosterone, dehydroepiandrosterone sulfate (DHEAS), hirsutism, sex hormone-binding globulin (SHBG), total antioxidant capacity (TAC) and malondialdehyde (MDA) levels.	Iran, 2021 [55]
Chronic kidney disease	Randomized crossover clinical trial of coenzyme Q10 and nicotinamide ribosome (NR) in chronic kidney disease	1200 mg/day for 6 weeks, per os	Six-weeks of treatment with NR or CoQ10 improved markers of systemic mitochondrial metabolism and lipid profiles but did not improve VO2 peak or total work efficiency. CoQ10 increased free fatty acids and decreased complex medium/long chain triglycerides.	USA, 2023 [56]
Diabetic neuropathy	Coenzyme Q10 as a potential add-on treatment for patients suffering from painful diabetic neuropathy: results of a placebo-controlled randomized trial	100 mg every 8 h for 8 weeks, per os	This trial support the idea that diabetic patients suffering from painful diabetic neuropathy may benefit from using antioxidant and anti-inflammatory supplements like CoQ10. However, further studies are required before supplementation with CoQ10 can be recommended for treating painful diabetic neuropathy.	Iran, 2021 [57]

## Data Availability

Not applicable.
